# Immune Toxicities in AAV Gene Therapy: Overview for Clinicians

**DOI:** 10.3390/ijms27073196

**Published:** 2026-03-31

**Authors:** Shestruma Parajuli, Thomas Gallagher, Terence R. Flotte

**Affiliations:** 1 Virginia Tech Carilion School of Medicine, Roanoke, VA 24016, USA; shestruma@vt.edu; 2 Department of Genetic and Cellular Medicine, UMass Chan Medical School, Worcester, MA 01655, USA; thomas.gallagher@umassmed.edu

**Keywords:** adeno-associated virus (AAV), gene therapy, immunotoxicity, immunomodulation, innate immunity, adaptive immunity, neutralizing antibody

## Abstract

Gene therapy using recombinant adeno-associated virus (rAAV) vectors has emerged as a transformative therapeutic modality for genetic disorders, demonstrating high transduction efficiency and a generally favorable safety profile during pre-clinical development. However, serious adverse events, including thrombotic microangiopathy, acute respiratory distress syndrome, hepatotoxicity, myocarditis, cytokine storm, and hemophagocytic lymphohistiocytosis, have been observed across multiple gene therapy clinical trials. Significant efforts have been made to understand the toxicities that cause these adverse events and clinical care for patients receiving gene therapies has evolved to mitigate their effects. These toxicities arise from a complex interplay between the innate and adaptive immune responses directed against the viral capsid and transgene products and are often compounded by pre-existing anti-AAV immunity. Immunomodulatory strategies have been developed to combat these responses to improve the long-term success of gene therapies, and this review provides clinicians managing gene therapy patients with an overview of mechanisms underlying AAV-associated immunotoxicities and a discussion of syndromes and mitigation strategies that have been reported in the clinical care of patients.

## 1. Introduction

Gene therapy has rapidly emerged as a transformative therapeutic modality for treating genetic disorders, with recombinant adeno-associated virus (rAAV) being widely used as a vector due to its favorable safety profile and high efficiency of transduction. rAAV vectors are engineered to be replication-incompetent by removing their viral coding sequences and were initially thought to elicit only weak inflammatory responses, primarily to the capsid and transgene cassette, giving them an appealing safety profile [1]. However, immunotoxicities have been reported following rAAV administration, especially very high-dose systemic delivery (>3 × 10^13^ vg/kg) [2,3]. Seven AAV-based therapies have received market authorization from the U.S. Food and Drug Administration (FDA): onasemnogene abeparvovec (Zolgensma) for Spinal Muscular Atrophy (SMA), voretigene neparvovec (Luxturna) for Inherited Retinal Dystrophy caused by *RPE65* mutations, valoctocogene roxaparvovec (Roctavian) for Hemophilia A, etranacogene dezaparvovec (Hemgenix) for Hemophilia B, fidanacogene elaparvovec (Beqvez) for Hemophilia B, eladocagene exuparvovec (Kebilidi) for Aromatic L-amino acid decarboxylase (AADC) deficiency, and delandistrogene moxeparvovec (Elevidys) for Duchenne Muscular Dystrophy (DMD) (Table 1) [4].

Immune responses and toxicities to rAAV therapies have emerged post-administration during clinical trials and post-market authorization, which manifest clinically as diagnosable syndromes with effects ranging from mild symptoms to fatal outcomes. Many of these immune responses and toxicities were not anticipated because they were not observed in animal models during pre-clinical testing, and their causes and mechanisms of action have only recently been investigated [5]. Thus, the translation of gene therapies from pre-clinical technologies into new genetic medicines must incorporate clinical care protocols to identify treatment-emergent syndromes in the days, weeks, months, and years following delivery to mitigate their harm and improve safety and efficacy as well as work to better understand the risks that contribute to their occurrence.

A significant complication for the clinical care of gene therapy patients and anticipating treatment-related toxicities is that the therapies are generally developed to treat rare genetic diseases, which are small populations of patients with confounding health burdens. Many rare disease patients have pre-existing sequelae of their disease at the time of gene therapy, such as cellular, tissue or organ dysfunction or inflammation, which may increase the risk of immunotoxicities and adverse outcomes. Thus, clinical monitoring and management is difficult to standardize across patient groups and must consider factors such as the biology and progression stage of the disease, age and characteristics of the patient, and design, dose, and route of administration of the vector. This review will discuss common mechanisms of immune toxicity in gene therapy, the syndromes that have been described in the clinic post-treatment, and contexts for increased risk and intervention strategies in the effort to advance clinical care for patients receiving gene therapies.

## 2. Pre-Existing Immunity and Routes of Administration

**Figure 1:**
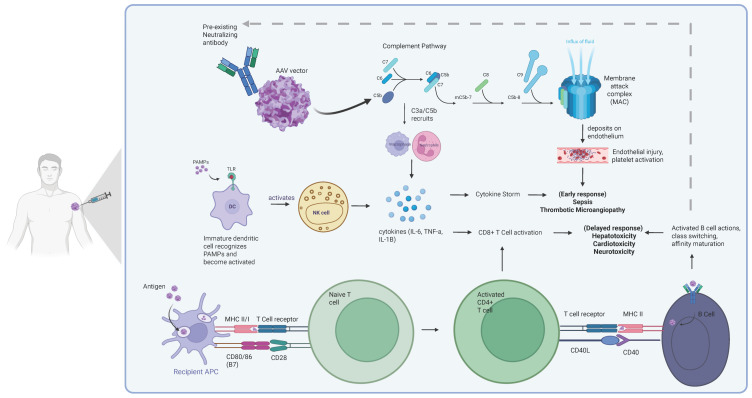
Major mechanisms of immune system activation to gene therapy. Created in BioRender. Thomas Gallagher (2026) https://BioRender.com/azhti25 (accessed on 17 February 2026).

The human immune system can be divided into innate and adaptive responses (Figure 1). Innate immune responses, including the complement system, occur early after encountering an antigen and do not result in immunological memory. Adaptive immune responses, however, are conditioned by previous inflammatory responses to an antigen and rely on the immunological memory, activation, and clonal expansion of differentiated antigen-specific B and T cell populations [6]. The rAAV capsids used to deliver gene therapies are derived from naturally occurring AAVs which are able to infect human populations, resulting in 30–60% of patients typically having pre-existing immune responses to the gene therapy product, although this can be as high as 100% in some populations [7,8]. Patients have different levels of neutralizing antibodies (NAbs) for each AAV serotype and the prevalence of NAbs for AAV serotypes varies geographically, but older patients have generally been found to have higher levels of Nabs [9,10]. High levels of pre-NAbs to AAVs in the circulation is a risk for both inhibiting transduction of host cells and causing toxicity. The prevalence of NAbs to the capsid of a specific AAV serotype can be screened for prior to enrolling patients in a clinical trial and may be used as exclusion criteria [11].

The impact of NAbs on gene therapy vectors was first evidenced in the AAV2-Factor IX (*FIX*) liver gene transfer trial, where two subjects with pre-existing NAbs received the highest dose (2 × 10^12^ vg/kg) of the vector [2,12]. The study showed that a NAb titer to AAV2 of 1:2 allowed for high expression levels of the FIX transgene, whereas a NAb titer of 1:17 prevented production of any detectable levels of circulating FIX following vector administration [13]. A study conducted by Smith et al. found that high AAV NAb titers (>1:100) enhanced the uptake of AAV viral particles into immune cells such as neutrophils, monocyte-related dendritic cells, and monocytes, leading to increased downstream production of pro-inflammatory cytokines and complement activation [14]. Mechanistically, NAbs bind to the capsid to prevent viral particles from entering the cells and lead to the initiation of the classical complement pathway. This humoral response leads to downstream B cell maturation, expansion, and class switching to increase antibody production against AAV particles.

The risk of immune system complications depends on the route of administration, which is generally either systemic delivery through intravenous (IV) injection or delivery to a targeted compartment or tissue through direct injection. Systemic delivery is less invasive, but results in widespread exposure of the capsid and transgene to immune components. Detection of the viral vector triggers a complex cascade of both innate and adaptive immune responses, which results in tissue damage and an active response to destroy the perceived pathogen and infected cells. This neutralizes the therapy and can cause severe toxicity. Systemically delivered gene therapy products accumulate in the liver following systemic delivery, which can cause hepatotoxicity progressively leading to liver failure, and so liver-sparing vector designs have been developed. These include engineering the capsid to reduce liver tropism and designing the transgene to be expressed from a tissue-specific promoter and/or be silenced by miRNAs expressed in hepatocytes, which can make therapies more efficient and lower the dose being delivered [15,16].

Direct injection reduces exposure of the gene therapy product to immune system components and peripheral tissues as well as lowers the therapeutic dose being delivered, but requires sophisticated technical expertise and can damage the tissue. The eye is relatively immune-privileged, and so there is a low risk of immune-related complications for AAV-based therapies delivered by local subretinal injection. The first AAV-based gene therapy to receive regulatory approval in the United States was Luxturna for the treatment of biallelic *RPE65* mutation-associated retinal dystrophy, which is delivered by subretinal injection. No deleterious immune responses have been reported, and the therapy could be successfully re-administered to both eyes because the initial treatment did not induce formation of NAbs, which is likely due to the immune privilege of the eye [17,18]. The subretinal space has limited exposure to plasma proteins, circulating antibodies, blood cells, and other immune cells, which prevents immune responses from being stimulated by the delivery of viral particles. However, age- and sex-specific differences in inflammatory responses have been reported following intravitreal injection [19].

Delivering gene therapies directly to the central nervous system (CNS) through routes such as intrathecal (IT), intra-cisterna magna (ICM), or intracerebroventricular (ICV) injection can increase transduction of CNS tissues, but can cause toxicity in the dorsal root ganglia (DRG), which manifests as ataxia and impaired ambulation [20,21,22,23]. DRG toxicity has been found to result from transgene overexpression and immune system activation, and so vector design strategies to better control transgene expression and immunosuppressive protocols to dampen inflammatory signaling and cytokine production are being developed. Delivery of vectors directly into the CNS largely, but not entirely, avoids encountering pre-existing NAbs and stimulating the adaptive immune response [24,25,26]. Intramuscular (IM) delivery is another common route of administration which has been utilized to concentrate expression of transgenes in muscle tissue to treat muscular dystrophies and/or act as durable biomanufacturing sources for the transgene product [27,28].

## 3. Adaptive Immune Response

Adaptive immune responses specific to the delivered rAAV capsid have been seen in most patients following systemic delivery. Adaptive immunity consists of humoral immunity, which acts through antibody-producing B cells, and cellular immunity, which acts through cytotoxic CD8+ T cells. Humoral immunity is primarily mediated by the production of antibodies against specific antigens. Naïve B cells first differentiate into short-lived plasma cells, which produce IgM antibodies. These antibodies undergo affinity maturation and class-switching, followed by differentiation of B cells into long-lived plasma cells and memory B cells [29], which produce antibodies that have increased responses to foreign particles and bind with increased avidity to their target antigen, which could be the rAAV capsid, transgene, and/or transgene product.

### 3.1. Anti-Capsid Responses

Many people have pre-existing antibodies to elements of rAAV capsids resulting from mother-to-child transmission and/or exposure to naturally occurring AAVs earlier in life, and dosing with AAV vectors results in the production of antibodies specifically to the rAAV [30]. Not all antibodies that can bind to rAAV capsids result in neutralization (i.e., NAbs are a subset of the total anti-rAAV antibodies), and specific assays for total and neutralizing anti-AAV antibody assays and their significance in the context of human gene therapy trials were recently reviewed thoroughly by Schulz et al. [8]. High levels of pre-existing antibodies that can bind to an rAAV capsid significantly reduces therapeutic efficacy and safety, and the generation of antibodies specifically to the delivered rAAV capsid severely limits re-dosing strategies to boost efficacy, which is one of the major hurdles to AAV-mediated gene therapy [2,31,32]. Thus, strategies to combat pre-existing NAbs to AAV capsids and disrupt the formation of rAAV capsid-specific antibodies are being developed in clinical protocols.

Anti-rAAV capsid responses have been observed in nearly every human trial of rAAV gene therapy, including the first clinical trial of aerosolized rAAV redosing trial for cystic fibrosis, which was found to be safe and well-tolerated but resulted in the development of serum AAV2-Nabs [33]. Similarly, a trial of an AAV2-alpha-1 antitrypsin vectors showed an increase in total binding antibody to AAV2 [34]. In another trial, Brantly et al. administered rAAV vectors packaged in an AAV1 capsid via IM injection at doses of 6.9 × 10^12^, 2.2 × 10^13^, and 6.0 × 10^13^, and then monitored immune responses using interferon (IFN)-γ enzyme-linked immunospot (ELISPOT) assays, lymphocyte proliferation assays, and serum antibody measurements against AAV1 capsid [35]. One remarkable finding of this study (shown in the manuscript’s Supplemental Information) was that large increases in serum NAb titer against distantly related serotypes, such as AAV7 and AAV8 were observed after IM administration of AAV1. The study found that anti-AAV1 capsid immune responses, including both NAbs and IFN-γ T cell response, developed by day 14. However, despite these responses, transgene expression was sustained at low but measurable levels for at least one year in the highest dose cohort, indicating that the immune response did not eliminate the transduced myofibers.

A study by Manno et al. also demonstrated contributions of cell-mediated immunity to immune-mediated toxicity [13]. Hemophilia B patients received infusion of a vector into the hepatic artery at doses up to 2 × 10^12^ vg/kg, which achieved therapeutic levels of FIX expression without acute or long-term toxicity [36]. This trial was the first to demonstrate that it is possible to transduce the human liver with AAV vectors to produce therapeutic levels of transgene expression. However, despite the initial success, the therapeutic levels of FIX gradually declined over time, which was accompanied by a transient asymptomatic elevation of liver transaminases, alanine aminotransferase (ALT) and aspartate aminotransferase (AST), as well as the emergence of AAV capsid-specific CD8+ T cells as indicated by INF-γ ELISPOT analysis, suggesting immune-mediated destruction of transduced hepatocytes. This response was determined to be directed against the AAV capsid rather than the wild-type *FIX* transgene due to recognition of a Major Histocompatibility Complex (MHC) Class I binding peptide. This adaptive immune response was not seen in animal models, including non-human primate (NHP) studies, emphasizing that there are challenges in human applications of gene therapies that cannot be predicted in pre-clinical studies.

Mingozzi et al. extended these findings by showing that human recipients of AAV vectors often harbor pre-existing memory CD8+ T cells that are specific to capsid epitopes, which has not been observed in animal models [37]. Furthermore, patients treated with the AAV vector infusion were shown to have consistently negative interleukin (IL)-5 responses, suggesting a cytotoxic response with activation of CD4+ and CD8+ rather than a Th2-driven immune mechanism. The study showed no pre-existing capsid-specific T cell responses at baseline, but IFN-γ producing cells were present at 2 weeks post-infusion. Following these initial findings in the AAV2-FIX trial, monitoring of capsid-directed T cell responses has been implemented in other muscle gene transfer clinical trials [38], which found a dose-dependent relationship between the vector and the activation of capsid-specific T cell responses. This indicates possible reactivation of memory T cells, highlighting the need for developing a comprehensive immunosuppression regimen that is effective for mitigating innate immune responses as well as the generation of antibody-producing B cells and cytotoxic T cells. Nathwani et al. subsequently showed that both the transaminase elevation and the suppression of transgene response were reversible with systemic corticosteroids [39]. The study demonstrated that patients who received a high dose of AAV8-FIX vector developed transient, asymptomatic increases in ALT post-infusion, which were temporally associated with declining FIX levels. This was consistent with an immune-mediated response against AAV capsid antigens presented by transduced hepatocytes, leading to cytotoxic T cell-mediated hepatocellular injury and loss of transgene expression. However, both transaminitis and transgene suppression were reversed with corticosteroid treatment, which represents the clearest and most consistent anti-vector response and indicates a direct effect of steroid therapy on suppressing these responses. The study also demonstrated that although elevated ALT levels were observed after vector infusion only in the high-dose group, an increase in the number of AAV8 capsid-reactive T cells were detectable in both intermediate- and low-dose groups. This underscores the importance of monitoring and potentially modulating immune responses in human AAV gene therapy trials to achieve durable transgene expression. Clinically, these findings have established corticosteroids as the first-line therapy for treating immune-mediated transaminitis in AAV gene therapy and guided protocols for immunosuppressive management to optimize long-term efficacy and safety.

### 3.2. Anti-Transgene Responses

Adaptive immunity has been shown to target the transgene rather than the capsid, which has been found to be influenced by factors such as the design of the transgene cassette, biology of the disease being treated, causative mutation, route of vector administration, target tissue, specific properties of the vector, and dose being delivered. Anti-transgene antibodies have been less commonly observed than anti-capsid, only consistently occurring in treatments for Pompe disease (deficiency of acid alpha-glucosidase, GAA), which has commonly been associated with anti-GAA antibodies being observed in patients who did not produce the wild-type copy of the gene (i.e., whose immune systems were naïve to the endogenous protein). The treatment strategy for Pompe disease was a single administration of an rAAV1-CMV-hGAA vector delivered by IM injection into the diaphragm at doses 100× lower than the maximal dose used in murine biodistribution studies. The study found that all subjects had anti-transgene antibody responses except for those who received concomitant immunomodulation with anti-CD20 monoclonal antibody (rituximab) and sirolimus. While anti-transgene T cell responses were detected, these responses did not result in clinically significant adverse events or loss of transgene expression [40].

Anti-transgene T cell responses have also been reported by Bonnemann et al. in patients receiving AAV-dystrophin vectors expressing portions of the dystrophin (*DMD*) gene that are deleted by the disease-causing mutation [41]. The study reported unexpected adverse events from three trials where different microdystrophin transgenes under different promotors were packaged in different AAV serotypes (AAV9, AAV8, AAVrh74) and delivered intravenously at doses 1 × 10^13^ and 2 × 10^14^ vg/kg. Adverse events included muscle weakness, myositis with increased creatine kinase (CK), elevated troponin, and muscle edema, along with T cell infiltration observed in muscle biopsy samples performed on two patients. Use of various immunosuppressive treatments such as glucocorticoids, IV immunoglobulin, plasmapheresis, and tacrolimus led to resolution of symptoms within 3 months. The timing of these adverse events was consistent with the induction of transgene expression, and the findings suggested a cytotoxic T cell immune response against dystrophin. This further highlights the immune complexities related to AAV gene therapy and the role of immunomodulators in enhancing the safety and efficacy of viral vector treatment.

## 4. Innate Immune Response

Innate immunity constitutes the initial and foremost defense mechanism of the human body to pathogens, and AAV gene therapies were initially thought to elicit relatively mild innate immune reactions compared to other viruses. The components of the innate immune system include physical barriers such as skin, specialized cells such as macrophages and dendritic cells, cellular receptors such as Toll-like receptors (TLRs), and soluble mediators such as cytokines and complement proteins [42]. Several clinical syndromes associated with innate immune responses to high-dose IV AAV gene therapy have been described (Table 2). 

In contrast with the manageable adaptive immune responses, innate responses to rAAV products have been associated with severe adverse events and deaths both in clinical trials and after receiving regulatory approval. Major immunological reactions involving both innate and adaptive responses against the capsid or transgene products pose a significant barrier to further progress and more widespread use of rAAVs, and can eventually lead to adverse effects such as hepatotoxicity [59], cardiotoxicity, thrombotic microangiopathy, and endothelial injury [3,29,60].

### 4.1. Thrombotic Microangiopathy (TMA)

The innate immune system also consists of the complement system, which can be activated by classical, lectin, and alternative pathways. The complement system plays an integral role in the immune system, detecting and eliminating pathogens as well as removing pathogen-immune complexes [42]. The classical pathway is activated by antibodies bound to antigen targets, while the alternative pathway is activated by interactions of foreign particles with the circulating complement components and therefore does not involve preformed antibodies. Administration of AAV vectors is known to induce highly potent *de novo* generation of IgM and IgG antibodies, thereby leading to classical complement pathway activation [42]. However, all complement pathways result in the formation of the C3 convertase, leading to the creation of the C5b-9 membrane attack complex (MAC) which induces cell lysis [42]. Activated complement pathway signaling initiates an inflammatory reaction, serving as a neutrophil attractant, triggering the coagulation pathway, activating platelets, and damaging endothelial cells [29]. Studies have shown that enzymatic degradation of IgG could potentially prevent complement-related immunotoxicity; however, the presence of IgM still causes significant immunotoxicity [42].

The mechanisms of immune-mediated toxicity were investigated in a study by Salabarria et al. linking complement-mediated thrombotic microangiopathy (TMA) to high-dose rAAV exposure in both clinical trials and approved therapies like Zolgensma (the lowest reported dose that has triggered TMA is 1 × 10^13^) [54]. TMA usually occurs 1–2 weeks post-dosing and is characterized by hemolytic anemia, thrombocytopenia, and microthrombi formation, and in cases where it is associated with renal toxicity, it would meet the definition of atypical hemolytic uremic syndrome (aHUS). It was shown that both the antibody-mediated classic pathway activating the complement system, as well as the direct binding of AAV may have contributed to this adverse effect. The study demonstrated that patients receiving rituximab and sirolimus showed suppressed antibody formation, minimal complement activity, and no evidence of TMA compared to patients receiving corticosteroids only, who developed increases in IgM and IgG along with an increase in D-dimer, a decline in platelet count, and indications of complement activation, highlighting the necessity of proper immune modulation strategies in mitigating AAV-related TMA. Salabarria et al. further demonstrated that pathogen-associated molecular patterns (PAMPs) and TLRs play a role in activating the innate immune system during microbial infections. PAMPs of rAAVs are primarily recognized by TLR2 and TLR9, which are highly expressed by innate immune cells such as macrophages and dendritic cells, initiating a type I IFN-driven inflammatory cascade that promotes CD8+ T cell activation [29]. The IL-1 receptor is present on the surface of a variety of cells, including endothelial cells, hepatocytes, fibroblasts, monocytes, neutrophils, T cells, and B cells. IL-1a and IL-1b bind to the IL-1 receptor to initiate a cascade of downstream signaling, leading to the activation of Nuclear Factor-κB (NF-κB) and p38 mitogen-activated protein kinase (MAPK) pathways and inducing secretion of pro-inflammatory cytokines [61,62]. In the context of AAV gene therapy, this initial innate immune signaling cascade could serve as an important target to mitigate innate immune response-mediated inflammation as well as the downstream adaptive immune response [1,42]. In a study by Li et al., encouraging results were seen after blocking TLR9 and/or IL-1 receptor signaling, which substantially reduced the CD8+ T cell response following systemic delivery of a low dose of vector. However, this strategy was ineffective when higher doses of vectors were delivered, which are required for many systemic dosing gene therapy strategies [63].

### 4.2. Hemophagocytic Lymphohistiocytosis (HLH)

Another case of immune toxicity was reported in a patient with SMA within 36 h after infusion of Zolgensma at a dose of 1.1 × 10^14^ vg/kg. The patient was reported to have Hemophagocytic Lymphohistiocytosis (HLH) caused by exaggerated responses of the immune system leading to hypercytokinemia [40]. HLH is a rare immunological condition that can occur secondary to uncontrolled, self-perpetuating activation of cytotoxic lymphocytes and macrophages. This leads to hyperproduction of pro-inflammatory cytokines such as IFN-γ, IL-1β, and IL-18 leading to tissue injury and multi-organ dysfunction, and laboratory findings involved pancytopenia, liver dysfunction, hypertriglyceridemia, hypofibrinogenemia, and elevated ferritin. In an SMA trial, symptoms consistent with HLH, such as fever, rash and hepatosplenomegaly, were noted and found to have resolved after treatment with the IL-1 receptor antagonist anakinra.

Another recent case of rAAV-associated HLH was described in a patient with Rett syndrome receiving a high dose (1.5 × 10^15^ vg/kg, total of 3 × 10^15^ vg) of rAAV9-MECP2 delivered by ICV injection. The patient had received a prophylactic immunomodulation regimen, which included steroids, sirolimus, and rituximab, but developed fever, vomiting, somnolence, cough, and poor oral intake one day after discharge. Notable laboratory findings, including markedly elevated ferritin, liver enzymes, and lactate dehydrogenase, pancytopenia, and coagulopathy, were seen with rapid clinical decline accompanied by respiratory failure, acute kidney injury, and hypotension. The patient was not reported to have clinical signs of TMA, in which thrombocytopenia is the most prominent feature. Despite the treatment with dexamethasone, anakinra, and a single dose of eculizumab, the multi-system failure was too advanced to stop clinical deterioration [64].

### 4.3. Endothelial Injury and Capillary Leak Pathology

A study published in the *New England Journal of Medicine* identified respiratory failure as a cause of death of a DMD patient following delivery of a customized high-dose systemic AAV gene therapy using an rAAV9 vector carrying dSaCas9 (a catalytically inactive form of Cas9 derived from *Staphylococcus aureus*) fused to the VP64 transcriptional activator (dSaCas9VP64), which was designed to upregulate cortical dystrophin expression [65]. The patient was a 27-year-old male with 30 kb deletion mutation at the 5′ end of the *DMD* gene who presented with loss of ambulation, progressive decline in arm function, and increasing cardiopulmonary dysfunction (36% of predicted value for Forced Expiratory Volume in 1 Second (FEV1) and forced vital capacity (FVC), and a left ventricular ejection fraction (LVEF) of 55–60%). Immunologic screening at baseline revealed a total antibody level against AAV9 below detection and showed negative ELISPOT responses to AAV9 and dSaCas9VP64. Prophylactic immune suppression therapy included rituximab beginning 13 days before administration of therapy, glucocorticoids 1 day before administration, and sirolimus 1 day before administration, which was delivered to the patient systemically at a dose of 1 × 10^14^ vg/kg. Within days of treatment, the patient developed progressive thrombocytopenia, respiratory acidosis, and elevated B-type natriuretic peptide, along with worsening cardiac function with a decline in LVEF to 45–50% and a troponin level of 0.59 ng. Pericardial effusion with characteristics of tamponade, along with acute respiratory distress syndrome (ARDS), eventually culminated in cardiopulmonary arrest and multi-organ failure. Treatment with glucocorticoids, eculizumab (C5 inhibitor), tocilizumab (IL-6 antagonist), anakinra (IL-1 receptor antagonist), and intravenous immunoglobulins was unsuccessful in halting the progression of multi-system dysfunction, and the patient died on day 8 post-treatment. Laboratory analysis revealed an elevation in IL-6 on day 5 post-treatment, along with a mild elevation in SC5b-9, suggesting insufficient complement blockage to a level of 453 ng/mL at 3 days after therapy. Elevation of IL-8 in the serum and high levels of Monocyte Chemoattractant Protein-1 (MCP-1) were also noted in a sample of pericardial fluid obtained 3 days post-therapy. IL-6 levels in the pericardial fluid were noted to be 100× higher than the IL-6 levels in the serum. The autopsy revealed decreased muscle mass in both heart and skeletal muscle as well as severe cardiomyopathy with fibrofatty replacement of biventricular myocardium. The lungs were edematous with diffuse alveolar damage characterized by hyaline membrane formation along with interstitial and interalveolar edema. Examination of the brain showed infarction in the cerebral cortex and cerebellum. No signs of complement deposition or TMA were detected. Furthermore, the absence of fibrin or complement deposition in the lung and heart indicated that complement-mediated TMA was not driving this toxicity. Cytokine-induced capillary leak syndrome was posited as the cause of the fatal immunotoxicity in this patient, although serum IL-6 elevations were also only slightly elevated at 2.8 pg/mL compared to the normal levels of <2 pg/mL. Host factors such as baseline serum cytokines may have also played a role in contributing towards respiratory insufficiency and cardiomyopathy due to relatively high vector transduction of muscle.

Further analyses of the samples from this patient have shown evidence of direct endothelial injury related to unexpectedly high levels of transgene expression in the endothelial cells of the lung [36]. Hordeaux et al. had observed a similar process in an NHP model [66] and, as with systemic AAV toxicity in humans, this form of toxicity was only seen at doses of IV-rAAV greater than 1 × 10^14^ vg/kg. The toxicity syndrome of transaminitis, thrombocytopenia, hypoalbuminemia, and activation of the alternative complement pathway was observed in a relatively high percentage of the animals. Histological and transcriptomic analysis further pointed to damage to liver sinusoidal endothelial cells (LSECs). Damaged LSECs exhibited elevated expression of endothelial activation markers such as von Willebrand factor (vWF) and CD31 (PECAM-1) along with decreased scavenger receptors and increased collagen production, indicating platelet sequestration and aggregation within hepatic sinusoids, further impairing liver microcirculation and amplifying hepatocellular injury. Although most animals recovered, some developed ascites, generalized edema, hyperbilirubinemia, and/or coagulopathy. Single-nucleus RNA sequencing (snRNA-seq) revealed robust AAV transduction in both parenchymal and endothelial liver cell types, with high transgene transcript levels observed early post-infusion. The study also indicated a correlation between thrombocytopenia and elevated serum hyaluronic acid (HA), reinforcing the role of endothelial barrier disruption in the observed toxicities. Direct comparison between NHP samples and the DMD patient mentioned above was performed using in situ hybridization (ISH) and snRNA-seq to investigate whether similar endothelial cell toxicity was present in the pulmonary endothelium. The findings showed clear ISH signal in the nucleus of hepatocytes with no signal in the cytoplasm, suggesting the presence of vector genome without active transcription. No ISH signal was detected in the heart, whereas endothelial cells of the lungs were strongly positive for both vector DNA and transcripts. The snRNA-seq data demonstrated high transgene transduction in the lungs and very low transduction in the liver and the heart. The study therefore concluded that systemic delivery of AAV9 can result in high transduction of pulmonary endothelial capillary cells, which has previously not been observed in NHPs or humans, and highlights the current gap in knowledge of the potential mechanisms causing immunotoxicities following systemic high-dose delivery of AAV gene therapy [36].

Another series of patients with LAMP2-deficiency (Danon disease) who experienced a similar toxicity after receiving IV AAV9 gene therapy was recently described by Jonathan Schwartz at a joint American Society of Cell & Gene Therapy (ASGCT)/Association of Regenerative Medicine (ARM) conference on 7 March 2026. In that study, patients had received prophylactic treatment with pegacetoplan, an FDA-approved complement inhibitor, and investigators suspect that pegacetoplan facilitated binding of a low concentration complement complexes to endothelial cells to cause diffuse capillary leak, which manifested as pulmonary edema and hemoconcentration.

Pre-clinical and clinical studies of immune-related toxicities such as TMA, HLH, and endothelial injury have implicated complement activation as one of the key pathogenic mechanisms responding to rAAV capsids, underscoring its critical role in host defense and highlighting it as a significant barrier to AAV therapies. Thus, clinical care strategies to mitigate the activation and/or effects of complement activation are being developed to improve the safety and efficacy of gene therapies.

### 4.4. Strategies to Mitigate Anti-Vector Immune Responses

Strategies aimed at modulating and mitigating immune responses, including addressing pre-existing immunity and limiting innate and adaptive immune reactions following vector administration, are critical for long-term therapeutic success and patient safety. However, no standard treatment regimen has been established and different combinations of prophylactic and symptom-responsive immunomodulation treatments have been administered. Clinical care decisions for each patient should be considered in the context of the immune mechanism which is being targeted and in consultation with sub-specialty teams who are knowledgeable about the disease being treated and organ systems that are affected (Table 3).

For mitigating innate immune responses, vector design strategies such as elimination or methylation of immunostimulatory CpG sequences in the transgene [67,68] as well as clinical management strategies such as administration of systemic corticosteroids, complement inhibitors, and monoclonal antibodies directed against specific innate immune cytokines [6,69] are being further explored.

For humoral immunity, agents that directly digest and eliminate circulating serum antibodies have been employed to reduce the level of pre-existing NAbs prior to dosing [70,71]. Anti-B cell therapies like rituximab (anti-CD20) have been used to prevent the development of new antibodies [72]. For combating cell-mediated immune responses, the use of corticosteroids and other general immunomodulatory agents have shown potential for extending the therapeutic benefits of gene therapy [73]. These agents may also aid with the dampening of humoral immune responses.

Ilifidase (IdeS) is an antibody cleaving agent that specifically cleaves circulating IgG antibodies, including anti-AAV antibodies, when infused intravenously. IdeS has been primarily used in kidney transplant patients, where it successfully reduced significant rejection or serious adverse events [74]. This ability to transiently eliminate circulating IgG was also seen in a study by Mingozzi et al. where IdeS reduced anti-AAV antibody levels in human plasma samples in vivo and prophylactic administration to NHPs successfully reduced circulating anti-AAV antibody levels and allowed for efficient gene transfer to the liver [70].

For cell-mediated immune responses, the use of corticosteroids has shown potential in extending the benefits of gene therapy. Agents such as prednisone, prednisolone, and methylprednisolone inhibit the expression of proinflammatory cytokines such as TNF-a, IL-6, and IL-1β, thereby limiting the downstream immune system activation. Pre-clinical and clinical studies have demonstrated that both prophylactic and reactive administration of corticosteroids are beneficial in preserving and enhancing transgene expression while limiting its adverse effects. These include reducing ALT levels in Hemophilia A, decreasing cytotoxic T cells, CD16+ monocytes, and dendritic cells in Hemophilia B, reducing IFN- γ and ALT levels in DMD, and reducing circulating lymphocytes in Tay-Sachs disease gene therapy trials [75].

Eculizumab is a complement C5 inhibitor that prevents the downstream formation of the Membrane Attack Complex (MAC), thereby reducing complement-mediated cell lysis and tissue damage. A case report from the University Children’s Hospital in Slovenia described a patient with SMA who developed TMA after a gene replacement therapy and was successfully treated with eculizumab. The patient initially presented with hemolytic anemia, thrombocytopenia, fragmented erythrocytes, increased AST, LDH, ferritin, and D-dimer, including signs of acute kidney injury and hemoglobinuria. Treatment with Eculizumab led to an improvement in both hematologic and renal parameters as well as inhibition of the complement system [76].

Cytokine inhibitors such as tocilizumab, an IL-6 receptor antagonist, and anakinra, an IL-1 receptor antagonist, are increasingly being used in gene therapy trials. Clinically, these agents are used in the weeks post-infusion in response to elevated inflammatory markers and symptomatic cytokine-driven toxicity, such as those seen in the study by Lek et al. [65]. A retrospective study with adults receiving Chimeric Antigen Receptor (CAR) T-cell therapy for cytokine release syndrome (CRS) compared the effectiveness of treatment using tocilizumab alone versus treatment with anakinra following an initial dose of tocilizumab, and both groups showed similar outcomes in the treatment of CRS [77]. Another study demonstrated similar findings with decreased cytokine levels following treatments with tocilizumab, representing an effective strategy to mitigate immune-medicated toxicities [78].

Calcineurin inhibitors such as tacrolimus are macrolide antibiotics which have immunosuppressive properties and inhibit calcium-dependent events, such as IL-2 gene transcription, nitric oxide synthase activation, and cell degranulation [79]. When administered prophylactically in a gene therapy study for hemophilia A, it led to a significant reduction in bleeding rates and increased overall FVIII expression. Additionally, when combined with prednisone, it further suppressed the proliferation of patient-derived activated CD8+ T cells in vitro [80]. Another study demonstrated similar findings where tacrolimus administration regulated the immune response to transgene and truncated microdystrophin in primates, highlighting its potential use in AAV-mediated gene therapy [81].

Sirolimus is a potent inhibitor of antigen-induced proliferation of T cells, B cells, and antibody production. It acts by forming an immunosuppressive complex with TOR, an intracellular protein that inhibits the activation of cell-cycle-specific kinase. In a study by Byrne et al. of SMA, DMD, GM1, and Danon disease patients receiving investigational or FDA-approved AAV9 gene therapies, prophylactic treatment with rituximab and sirolimus in addition to corticosteroids prevented anti-AAV antibody formation to a greater extent than corticosteroids alone [82]. However, not all acute phase AAV toxicities are due to adaptive immune responses. As one example, Lek et al. reported that prophylactic use of sirolimus did not prevent adverse effects in a patient with advanced DMD [65]. Prophylactic sirolimus administration was intended to prevent adaptive immune responses, and no adaptive immune responses were observed in this patient (i.e., no measurable anti-AAV antibody production or ELISPOT responses). Further analysis revealed that there was acute endothelial injury beginning 48 h after administration, associated with unexpected transgene expression in endothelial cells [36]. It possible that this could have been innate immune mediated or that endothelial transgene expression caused injury to endothelial cells without innate immune activation, or both mechanisms may have been active.

### 4.5. Alternatives to Viral Vectors

Many of the toxicities caused by viral vectors are due to the immune system recognizing pathogenic features of the viral particle and pre-existing NAbs formed by exposure to viruses earlier in life. Non-viral vectors such as lipid nanoparticles (LNPs) and N-acetylgalactosamine (GalNAc) have been developed as alternative delivery platforms to avoid these risks for toxicity and been used in approved gene therapy products for diseases such as human Transthyretin Amyloidosis (hATTR), Acute Hepatic Porphyria (AHP), Primary Hyperoxaluria Type 1, and Hypercholesterolemia. LNPs are typically composed of phospholipids, ionizable lipids, polyethylene glycol (PEG)-lipids, and cholesterol, but targeting moieties (e.g., peptides and antibodies) can be added to their exterior surface to improve specificity for a desired cell type. LNPs encapsulate their cargo, protecting it from exposure to immune system components, and are commonly used to deliver small interfering RNAs (siRNAs), messenger RNAs, anti-sense oligonucleotides (ASOs), and CPRISPR/Cas9 editing tools [83]. GalNAc moieties are conjugated to siRNA or ASO sequences and facilitate their delivery to liver cells following subcutaneous delivery through binding to the asialoglycoprotein receptor carbohydrate recognition domain, which is primarily expressed on the surface of hepatocytes [84]. Non-viral vectors offer reduced immunogenicity, higher cargo capacity, and more cost-efficient manufacturing production compared to viral vectors. However, non-viral vectors are susceptible to endonuclease degradation, have restricted biodistribution/tropism, and low transfection efficiency compared to viral vectors, which limits their utility as gene delivery platforms. Thus, while non-viral vectors have great potential to avoid the toxicities that viral vectors can induce, further development is needed to improve their tissue-targeting and gene delivery efficiencies to produce durable therapeutic effects.

## 5. Conclusions

This review highlights recent advances in AAV gene therapy with an emphasis on clinically observed immune toxicities, their underlying mechanism, and mitigating strategies. While early therapeutic successes have demonstrated the potential for AAV-based gene therapy as a new form of medicine, significant challenges remain to ensure their safety and efficacy. Major immunological reactions, driven by both innate and adaptive responses against the capsids and transgene products as well as NAbs, represent major barriers to broader clinical translation and long-term efficacy. Diagnosable syndromes have been reported across gene therapy trials and clinical care of these patients has advanced to anticipate and respond to treatment-emergent adverse events. However, further research is needed to identify risk factors for particular vector designs, diseases, and patients to establish prophylactic and responsive clinical care protocols that mitigate their effects. With continued research and improved understanding of the complex interplay between AAV vectors and host immune responses, clinical treatment strategies to combat immunotoxicities can help realize the potential for gene therapy to produce safer and more effective outcomes for patients.

## Figures and Tables

**Table 1:** FDA-approved gene therapy products.

Product	Gene Delivered	Vector Serotype	Disease Targeted	Approval Year
onasemnogene abeparvovec (Zolgensma)	*SMN1*	AAV9	Spinal Muscular Atrophy (SMA)	2019 (FDA)
voretigene neparvovec (Luxturna)	Retinoid isomerohydrolase *RPE65*	AAV2	Inherited Retinal Dystrophy due to *RPE 65* mutations	2017 (FDA)
valoctocogene roxaparvovec (Roctavian)	Factor VIII (*F8*)	AAV5	Hemophilia A	2022/2023 (FDA/EMA)
etranacogene dezaparvovec (Hemgenix)	Padua variant of Factor IX (*F9*)	AAV5	Hemophilia B	2022 (FDA)
fidanacogene elaparvovec (Beqvez)	Padua variant of Factor IX (*F9*)	AAVrh74var	Hemophilia B	2024 (FDA/EMA)
eladocagene exuparvovec (Kebilidi)	Dopa decardoxylase (*DDC*)	Modified rAAV2 capsid	Aromatic L-amino Acid Decardoxylase (AADC) deficiency	2024 (FDA/EMA)
delandistrogene moxeparvovec (Elevidys)	Micro-dystrophin	AAVrh74	Duchenne Muscular Dystrophy (DMD)	2023 (FDA)

**Table 2:** Clinical syndromes caused by acute reactions to systemic high-dose AAV gene therapy. * The emergence of post-administration syndromes requires further study, the mechanisms causing them may be multi-faceted and have not been definitively established.

Clinical Syndrome	Treatment Context	Clinical Manifestations	Proposed Mechanism of Action *	Frequency/Time of Onset Post-Infusion	Citations
Acute hepatotoxicity	Hemophilia, SMA, and very high-dose systemic administration	Elevated levels of transaminases (ALT and AST) in the circulation, progressing to liver if not treated. Lead to mortality in only 0.2% of patients receiving Zolgensma.	Direct toxicity from excess vector load, hepatocyte transgene expression, and/or TLR-activation as well as indirect immune-mediated toxicity	20–86%/1–3 weeks post-treatment	[43,44]
Myocarditis	Muscular dystrophies	Elevated troponin and creatine kinase levels, EKG abnormalities, decreased cardiac function	Direct toxicity from excess vector load, cardiomyocyte transgene expression, and/or TLR-activation as well as indirect immune-mediated toxicity	Three out of five patients in a clinical trial and 50% of NHPs in a pre-clinical study/3–6 weeks post-treatment	[41,45,46,47,48]
Thrombocytopenia	SMA and very high-dose systemic administration	Decreased platelet counts, bruising, bleeding	Platelet adherence to endothelial, sequestration in liver	75–90%/1 week post-administration	[49,50,51,52,53,54]
Cytokine Storm	High doses of IV rAAV	Multi-organ system dysfunction	TLR-mediated cytokine release	Not well established	[55]
Thrombotic Micro-angiopathy (TMA)	Pre-existing anti-capsid antibodies	Disseminated intravascular coagulation, with thrombocytopenia, elevated international normalized ratio, elevated fibrin split products	Complement activation through the classic or alternative pathway	1–2 weeks post-administration	[49,50,54,56]
Acute Respiratory Distress Syndrome (ARDS)	Muscular dystrophies	Diffuse capillary leak with pulmonary edema, diffuse alveolar injury and hyaline membrane formation, ascites, pleural and pericardial effusions	Direct endothelial injury from transgene over-expression	Not well established	[57]
Hemophagocytic Lymphohistiocytosis (HLH)	SMA and very high-dose systemic administration; and after ICV delivery	Fever, thrombocytopenia, hepatosplenomegaly, serum ferritin > 2200 ng/mL, sCD25 > 8800 U/mL, hemophagocytosis in bone marrow and lymph nodes	Excessive cytokine release	Not well established	[40,58]

**Table 3:** Immune modulators used in the clinical care of gene therapy patients.

Immune Modulator Class	Mechanism of Action	Treatment Context	Type(s) of Immunity Modulated
Antibody cleaving agents Example: Imlifidase	Cleaves circulating IgG antibodies	Prophylactic for patients with pre-existing NAbs	Humoral pre-existing
Corticosteroids Example: Prednisone, prednisolone, methylprednisolone	Inhibit expression of proinflammatory cytokines	Prophylactic and in response to elevated liver enzymes (AST, ALT, GGT), hepatotoxicity, rise in NT-proBNP, jaundice, and/or thrombocytopenia	Innate, humoral adaptive, cell-mediated adaptive
Complement inhibitors Example: Eculizumab	C5 inhibitor → inhibits formation of MAC	In response to TMA; hemolytic anemia, thrombocytopenia, fragmented erythrocytes, increased AST, LDH, ferritin, D-dimer, AKI, and/or hemoglobinuria	Innate, humoral activation of complement (classical)
Cytokine inhibitors Example: Tocilizumab, Anakinra	IL-6 receptor antagonist, IL-1 receptor antagonist, respectively	In response to elevated inflammatory markers such as IL-6, IFN-γ, TNF-α, IL-10, IL-1, IL-2, IL-5, IL-8, granulocyte-macrophage colony-stimulating factor, and serum biochemical markers, including CRP and ferritin	Innate
Calcineurin inhibitors Example: Tacrolimus	Inhibits calcium-dependent events	Prophylactic to prevent T-cell activation and IL-2 production	Cell-mediate adaptive, humoral adaptive
mTOR inhibitors Example: Sirolimus	Inhibits activation of cell-cycle-specific kinase, TOR	Prophylactic to prevent anti-AAV antibody formation	Cell-mediate adaptive, humoral adaptive

## Data Availability

No new data were created or analyzed in this study. Data sharing is not applicable to this article.
